# Recurrent aphthous stomatitis and Helicobacter pylori

**DOI:** 10.4317/medoral.20872

**Published:** 2016-01-31

**Authors:** Carolina-Cavaliéri Gomes, Ricardo-Santiago Gomez, Lívia-Guimarães Zina, Fabrício-Rezende Amaral

**Affiliations:** 1 Department of Pathology, Biological Sciences Institute, Universidade Federal de Minas Gerais, Belo Horizonte, Minas Gerais, Brazil; 2 Department of Oral Pathology, School of Dentistry, Universidade Federal de Minas Gerais, Belo Horizonte, Minas Gerais, Brazil; 3 Department of Social and Preventive Dentistry, Universidade Federal de Minas Gerais, Belo Horizonte, Minas Gerais, Brazil; 4 Dentist of the Oral Medicine Service of the Brazilian Army

## Abstract

**Background:**

Recurrent aphthous stomatitis (RAS) is a recurrent painful ulcerative disorder that commonly affects the oral mucosa. Local and systemic factors such as trauma, food sensitivity, nutritional deficiencies, systemic conditions, immunological disorders and genetic polymorphisms are associated with the development of the disease. *Helicobacter pylori* (*H. pylori*) is a gram-negative, microaerophile bacteria, that colonizes the gastric mucosa and it was previously suggested to be involved in RAS development. In the present paper we reviewed all previous studies that investigated the association between RAS and *H. pylori*.

**Material and Methods:**

A search in Pubmed (MEDLINE) databases was made of articles published up until July 2015 using the following keywords: *Helicobacter Pylori or H. pylori* and RAS or Recurrent aphthous stomatitis.

**Results:**

Fifteen experimental studies that addressed the relationship between infection with *H. pylori* and the presence of RAS and three reviews, including a systematic review and a meta-analysis were included in this review. The studies reviewed used different methods to assess this relationship, including PCR, nested PCR, culture, ELISA and urea breath test. A large variation in the number of patients included in each study, as well as inclusion criteria and laboratorial methods was observed. *H. pylori* can be detected in the oral mucosa or ulcerated lesion of some patients with RAS. The quality of the all studies included in this review was assessed using levels of evidence based on the University of Oxford’s Center for Evidence Based Medicine Criteria.

**Conclusions:**

Although the eradication of the infection may affect the clinical course of the oral lesions by undetermined mechanisms, RAS ulcers are not associated with the presence of the bacteria in the oral cavity and there is no evidence that *H. pylori* infection drives RAS development.

**Key words:**Campylobacter, elisa, h. pylori, Helicobacter Pylori, RAS, recurrent aphthous stomatitis, PCR.

## Introduction

**Figure 1:**
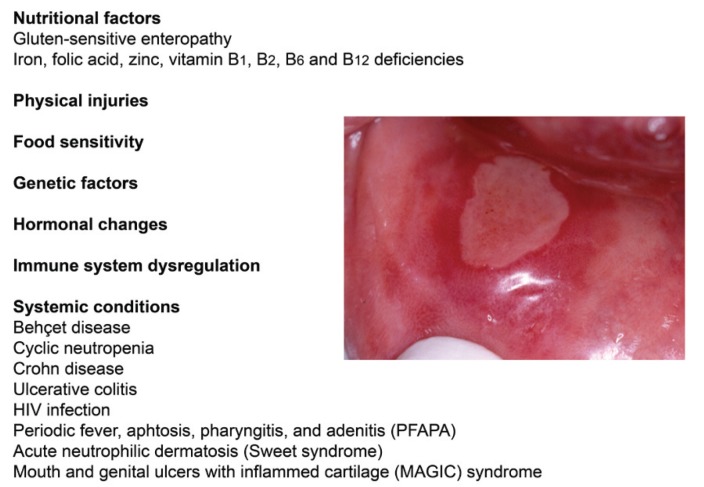
Clinical picture of a RAS lesion and the etiological factors associated with its development.

Recurrent aphthous stomatitis (RAS) is a very common condition characterized by solitary or multiple small, round, recurrent oral ulcers, with erythematous haloes and circumscribed margins. The appearance of the painful ulcers is periodic and the onset is usually during childhood and tends to diminish in severity with age (1). The diagnosis of RAS is based on clinical grounds but the etiology and pathogenesis remain unclear (2). Local and systemic factors have been suggested to affect the development of RAS. These factors are illustrated in the figure 1. For example, some genetic polymorphisms are associated with the occurrence of RAS (3). Some predisposing factors include trauma, hormonal changes, diet, nutritional deficiencies, Coeliac disease, and immunological disorders (4,5). Regarding nutritional deficiencies, some studies have found decreased levels of iron, vitamin B3 and B12, vitamin C, and folic acid (2).

*Helicobacter pylori *(*H. pylori*) is a gram-negative, microaerophile bacteria, that colonizes the gastric mucosa and its infection is associated with the development of peptic ulcers, gastric mucosa associated lymphoid tissue lymphoma, and gastric cancer (6). Although *H. pylori* infection has been suggested to be one of the etiological factors in the pathogenesis of RAS, this association is debatable. In the present paper we review this issue and present the available evidence regarding this controversial topic.

## Material and Methods

- Association between RAS and *helicobacter pylori*

In this review, a search in Pubmed (MEDLINE) databases was made of articles published up until July 2015 using the following keywords: *Helicobacter Pylori or H. pylori* and RAS or Recurrent aphthous stomatitis. We included experimental and review studies that assessed the relationship between *H. pylori* and RAS. Quality of the studies was assessed using levels of evidence based on the University of Oxford’s Center for Evidence Based Medicine Criteria (CEMB 2009) ( Table 1).

**Table 1:**
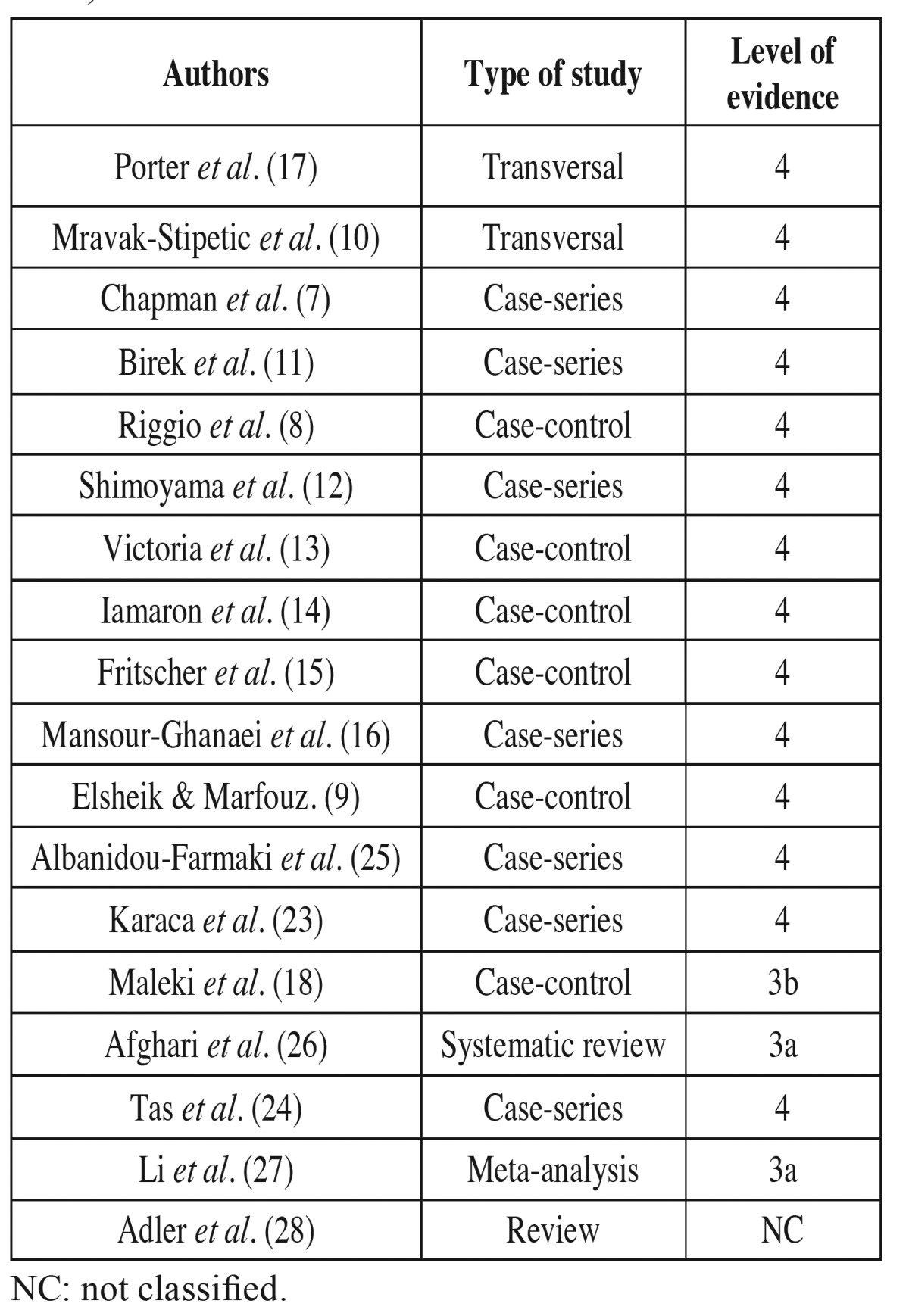
Classification of the studies selected for review according to type of study and level of evidence (CEMB 2009).

## Results and Discussion

We included in this review fifteen experimental studies that addressed the relationship between infection with *H. pylori* and the presence of RAS and three reviews, including a systematic review and a meta-analysis that assessed this association ( Table 2). As shown in  Table 2 there was a large variation in the number of patients evaluated in each study as well as the methods used to collect the samples or to identify *H. pylori*. While in some studies biopsies of the lesions were used (7-9), others used swabs (10-16). Ten out of fifteen studies did not demonstrate a statistically significant association between *H. pylori* and the presence of RAS (7,8,10,12-18). Another important variation that affects the analysis of the studies is the inclusion criteria used to diagnose RAS. As the histopathological features of RAS are nonspecific and the diagnosis is based on clinical grounds, the standardization of patients’ selection in future studies is important. There are good reviews about the clinical and diagnostic aspects of the disease (19).

**Table 2:**
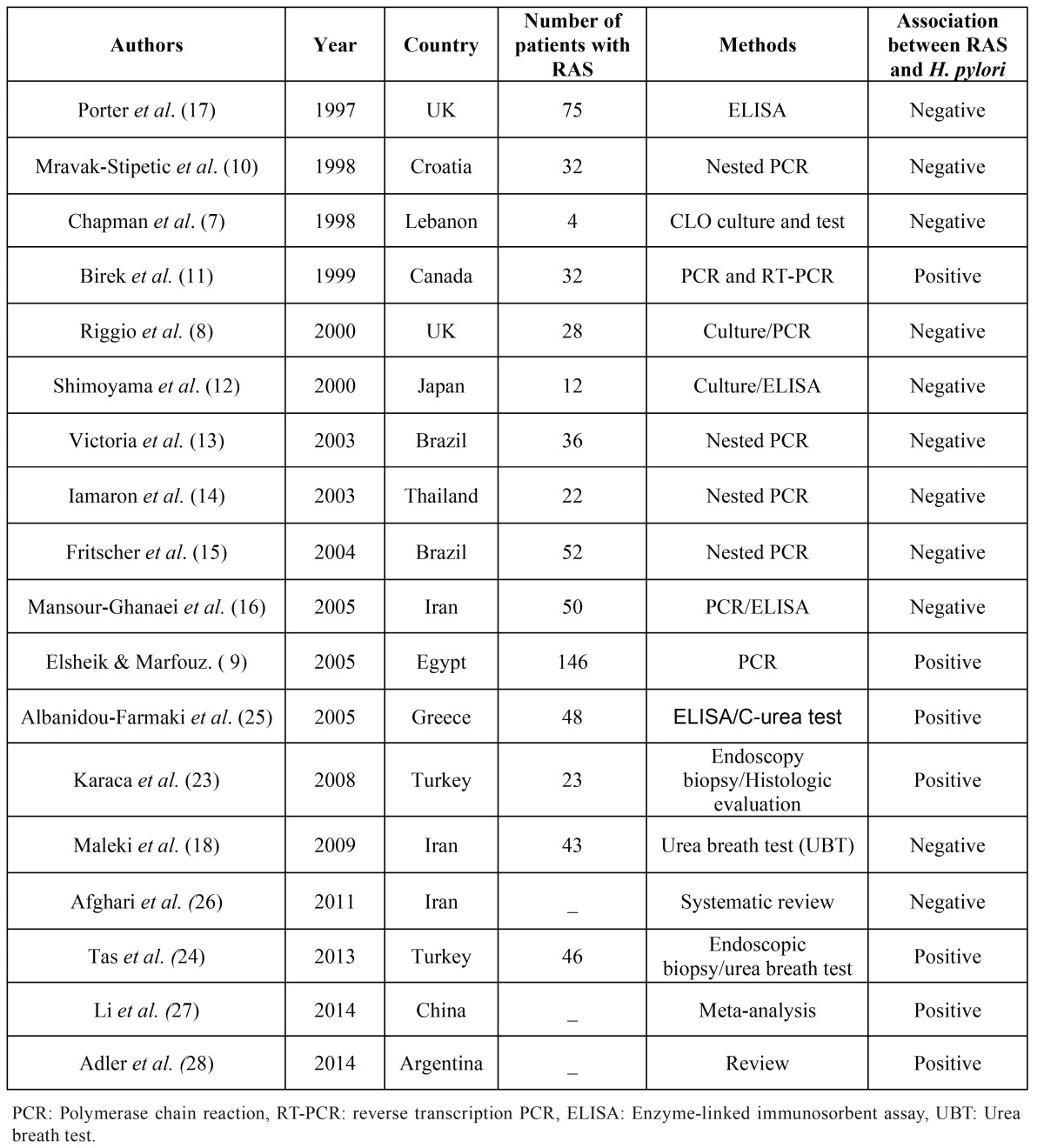
Studies that investigated the association between recurrent aphthous stomatitis (RAS) development and *H. pylori* infection.

The polymerase chain reaction (PCR) method was used to identify the presence of *H. pylori* DNA in eight studies (8-11,13-16). In two of them, the authors reported a statistically significant association between *H.pylori* presence and RAS (9,11). While Birek *et al*. (11) detected *H. pylori* in 72% of RAS samples using PCR and RT-PCR, Elsheikh & Mahfouz (9) reported that this was mainly observed in lesions localized in mucosa-associated lymphoid tissue of pharynx. However, it is necessary to emphasise that the simple detection of the bacteria in the oral lesion does not mean a causal relationship, as the microorganism may be a “passenger” and may not be the initiating factor of the disease. Most of the studies that employed PCR or nested PCR did not find association between the presence of the bacteria in the oral lesions and its development (8,10,13-16). *H. pylori* DNA was detected between 2% and 38.9% of RAS lesions included in the studies (8,10,13-16). None of these studies reported a statistically significant difference between the number of positive samples in the case and control groups. It is interesting that all authors who used the highly sensitive nested PCR method to detect the presence of *H. pylori* DNA in oral lesions did not find a positive relationship (10,13-15). The frequency of *H. pylori* in the non-affected oral mucosa of patients with RAS is not different from those without this condition (10,13-15).

Song *et al*. (20) compared three of the most used sets of primers for PCR analysis of *H. pylori* on samples of dental plaque. They concluded that the primer pairs EHC-U/EHC-L was the most recommended for the detection of *H. pylori* in the oral cavity. An important issue that should be addressed is that the PCR-based studies used different primers, some of which specific to conserved regions of the *H. pylori* genome, and some detecting the variable regions of the genome. The possible presence of other *Helicobacter-like* species in the mouth such as *Campylobacter Rectus, Campylobacter Curvus* and *Campylobacter Concisus* that are periodontopathogens and have up to 90% similarity with *H. pylori* is also a factor that needs to be considered (21). In addition, some of the primers used in the studies could also amplify other *Helicobacter* species that have also been found in human gut, such as *H. fennelliae, H cinaedias* (22). Thus appropriate positive and negative controls together with DNA direct sequencing of the PCR product are necessary to define the best PCR conditions and primers that should be used to detect *H. pylori* in samples collected from the oral cavity.

In two studies, patients with RAS were submitted to endoscopy biopsy to detect *H. pylori* (23,24). Both studies showed a positive relationship between the presence of the bacteria in the stomach and the occurrence of RAS in the mouth. In the studies of Karaca *et al*. (23) and Tas *et al*. (24) 87% and 65% of the patients with RAS, respectively, showed the bacteria in the gastric mucosa.

In four studies, the enzyme-linked immunosorbant assay (ELISA) was used to detect specific antibodies to *H. pylori* in RAS patients (12,16,17,25). In the study of Farmaki *et al*. (25) most of the patients with RAS were *H. pylori* positive in the ELISA test of serum and saliva. The other studies that performed ELISA did not find association between the presence of anti-*H. pylori* antibodies and RAS. Mansour-Ghanaei *et al*. (16) found that 26 (52%) of 50 subjects with RAS analysed were positive for *H. pylori* in the ELISA test. Of these, 16 patients had gastric disorders and 10 did not. These authors did not find a relationship between *H. pylori* positivity in the ELISA test and the presence of this bacterium in RAS lesions. In the study of Porter *et al*. (17) the frequency of anti-*H. pylori* seropositivity was not significantly greater in patients with RAS (30.6%) compared with patients with other ulcerated lesions (33.0%) and controls (24%). Shimoyama *et al*. (12) measured the presence of IgG antibody against *H. pylori* in the serum of 12 patients and found only three seropositive cases. Although no temporal analysis was performed in any of these studies, on the basis of these ELISA-based findings, there is no sound evidence of any association between the *H. pylori* infection and RAS.

Two studies used culture to detect *H. pylori* in RAS lesions. Shimoyama *et al*. (12) employed culture in samples collected by swabbing in the ulcer surface of 12 patients to detect *H. pylori*. Their results showed that none of 12 patients were positive for the bacteria in the culture. Chapman *et al*. (7) did not find the presence of the *H. pylori* in the biopsy samples obtained from patients with active RAS and history of RAS after performing CLO (*Campylobacter-like* organism) culture. In addition, no association between *H. pylori* and RAS was observed by Maleki *et al*. (18) using the UBT (Urea Breath Test). Furthermore, it is important to state that *H. pylori* in the oral cavity might be in a non-culturable coccoid state without the productive infection (12).

Recent literature review, including a systematic review and a meta-analysis, assessed the association between the infection by *H. pylori* and RAS. Afghari *et al*. (26) after reviewing nine publications up to 2011, concluded that there is no association. Li *et al*. (27) conducted a meta-analysis on studies published up to 2013 that evaluated the prevalence of infection by *H. pylori* in patients with RAS and controls. In this review they found an increased risk of RAS in patients with infection by *H. pylori* and eradication of this infection may prevent occurrence of RAS. Adler *et al*. (28) in a revision concluded that *H. pylori* infection in the occurrence of RAS would be associated with the anemia produced by *H pylori*-positive stomach disease.

- Helicobacter pylori eradication and RAS 

While there is no evidence that RAS ulcers are directly associated with the infection by *H. pylori*, some studies have demonstrated that the eradication of the bacteria affects the clinical course of the oral disease (23-25). Karaca *et al*. (23) studied 23 patients with RAS and performed endoscopy and gastric biopsies. The patients with *H. pylori* were put on an eradication therapy and follow-up for annual recurrence. They observed significant positive effect of eradication on the recurrence rate, number, diameter, and amelioration of time of RAS. Albanidou-Farmaki *et al*. (25) studied 34 RAS patients that were positive for *H. pylori*. After the eradication therapy, the group of patients who had become negative showed a remarkable improvement with respect to recurrence of RAS lesions and symptom intensity. Tas *et al*. (24) studied forty-six patients with RAS during 6 months and recorded vitamin B12 levels. Thirty of these 46 subjects were positive for *H. pylori*. They found that vitamin B12 levels were significantly increased in the group of *H. pylori*-eradicated RAS patients. In addition, the number of RAS lesions in these patients decreased significantly. This study suggests that vitamin B12 levels could be the underlying mechanism that explains the effect of *H. pylori* eradication on RAS development. However, these findings need to be further confirmed in a large group of patients with a long follow-up period. Furthermore, other biological mechanisms related to *H. pylori* infection and treatment should be investigated.

## Conclusion

The *H. pylori* can be occasionally detected in RAS lesions and the eradication of the infection may affect the clinical course of RAS lesions by undetermined mechanisms. However, most of the studies do not support the association of RAS ulcers with the presence of the bacteria in the oral cavity and the presence of the bacteria in the ulcer may reflect a passenger infection and not the trigger event. There is no convincing evidence of a direct cause- consequence effect of *H. pylori *infection and RAS ulcers development. This association requires further investigation by well-design prospective studies.The debate goes on.
